# Linguistic analysis of pediatric residency personal statements: gender differences

**DOI:** 10.1186/s12909-019-1838-x

**Published:** 2019-10-26

**Authors:** Jessica C. Babal, Aubrey D. Gower, John G. Frohna, Megan A. Moreno

**Affiliations:** 1 Department of Pediatrics, University of Wisconsin School of Medicine and Public Health, 2870 University Ave, Suite 200, Madison, WI 53705 USA; 2 Department of Pediatrics, University of Wisconsin School of Medicine and Public Health, Madison, WI USA; 3 Pediatrics Residency Program Director, Department of Pediatrics, University of Wisconsin School of Medicine and Public Health, Madison, WI USA; 4 Academic Division Chief, General Pediatrics and Adolescent Medicine, Department of Pediatrics, University of Wisconsin School of Medicine and Public Health, Madison, WI USA

**Keywords:** Linguistic analysis, Gender, Bias, Residency, Pediatrics

## Abstract

**Background:**

All US residency programs require applicants to submit personal statements. Prior studies showed gender differences in personal statement writing, which has implications for gender bias in the application process, but previous studies have not considered the dual influence of specialty-specific values on personal statement writing by applicants of each gender.

**Objective:**

To understand gender differences in pediatric residency personal statements.

**Methods:**

From 2017 to 2018, we performed linguistic analysis of personal statements written by interviewees at a mid-size US pediatrics residency during two prior academic years. We assessed writing tone, communal language, and agentic language. We performed t-tests to evaluate for gender differences, *p* < 0.05.

**Results:**

We analyzed personal statements from 85 male and 85 female interviewees. Average word count was 676 words. Personal statements demonstrated analytic writing style with authentic and positive emotional tone. We found no gender differences in communal language for social affiliation (*p* = 0.31), adjectives (*p* = 0.49), or orientation (*p* = 0.48), which deviates from typical gender norms for male language use. Males used agentic language of reward more frequently (*p* = 0.02).

**Conclusions:**

Findings suggest that social language is valued in pediatrics, a predominantly female specialty, regardless of applicant gender. Use of reward language by males is consistent with previous findings. Future studies should evaluate gender differences in residency applications across specialties to advance understanding of the role gender plays in the application process.

## Introduction

Gender disparities have been pervasive in medicine worldwide. Women historically have been admitted less frequently to medical school, paid less than men for comparable work, and promoted less often to leadership roles [1, 2]. In recent decades, women have made progress, representing more than half of all students admitted to US medical schools [3]. However, women practicing medicine continue to be paid less and promoted less than their male colleagues [2, 4–6].

Much of the persistent disparity may be attributed to gender biases [1, 7]. Gender biases, which are highly ingrained and often subtle, are driven by expectations of how people should behave or speak, based on assigned gender. In general, females are expected to use communal (social, relationship-oriented) language and behave in communal ways, while men are expected to use agentic (self-oriented) language and behave in agentic ways [7–12]. Communal language includes references to others (family, friend, colleague); language lacking self-assertiveness (perhaps, maybe); and relationship-oriented adjectives (affectionate, helpful). Agentic language includes psychological drives that promote the self (reward, risk, power, achievement); assertive language (think, know, always, never); and adjectives showing self-assuredness (confident, ambitious).

Numerous studies show that when women in particular fail to adhere to communal gender stereotypes in the workplace, they suffer professional advancement penalties [13–16]. These concerns are associated with many women altering their speech and behavior to avoid self-promotion, especially in specialties in which females represent the minority of the workforce or when performing traditionally “male” tasks [15, 17–19]. Less is known about how males working in female-dominated specialties, such as pediatrics, fare based on the language they use.

Previous studies show that gender bias begins early in a medical career, starting with the Medical Student Performance Evaluation (MSPE) and specialty-specific letters of recommendation, required for all applications to US residency programs [20–24]. In these performance evaluations and letters of recommendation, evaluators are more likely to describe male students using agentic standout words (e.g. “exceptional”) and as leaders with innate ability, whereas women are described as strong communicators, team members, and hard workers [21, 23, 24]. These gender biases have been shown to persist in application processes throughout the medical career, including in letters of recommendation for faculty positions and reviewer critiques for grant funding [25, 26].

While letters of recommendation provide insight into the ways that gender bias might shape faculty evaluations of the applicant, linguistic analysis of personal statements offers a different perspective in the application process—an understanding of the language and stories that applicants use to convey that they are qualified for the field to which they are applying. Notably, letters of recommendation and personal statements both have shown lack of inter-rater reliability and lack of correlation with future clinical performance [27–31]. Additional concerns have been raised about the utility of personal statements, as many see these essays as impersonal and lacking in authenticity [32]. Yet, most programs continue to use the personal statement in their residency selection process as a means of allowing students to demonstrate unique characteristics of their personality and “fit” within the medical specialty they have chosen to pursue [33]. Linguistic analysis of personal statements may therefore provide an important opportunity to optimize use of the personal statement in understanding the values and perspectives of residency applicants by its ability to detect subtle differences in applicant writing.

Studies that previously evaluated personal statements for gender differences in internal medicine and general surgery programs showed that applicants of both genders described the importance of agentic professional values, including appeal of problem-solving in internal medicine and technical skills in surgery, but applicants otherwise upheld gender norms for communality and agency [34, 35]. Females applying to medicine and surgery programs wrote more often about communal themes (interpersonal relationships and team-based care), and males focused more on agency (academic rigor, clinical excellence, and self-promotion) [34, 35]. These findings suggest that in writing their personal statements to residency, applicants may be subject to dual social pressures of simultaneously upholding gender norms for social and agentic language use, while also demonstrating shared professional values for communality and agency with the members of their anticipated profession.

To date, no studies have examined linguistic gender differences in personal statements for pediatric residency. This area of study is particularly intriguing because pediatrics has been one of the few predominantly female medical specialties and as such, may present unique gender expectations for its future clinicians. Additionally, the focus in pediatrics on family-centered care may attract medical students with a more communal focus in their approach to clinical care, such that this population may focus on these values more heavily than would be expected in personal statements submitted for other medical specialties. The purpose of this study was to evaluate the linguistic characteristics of pediatric residency personal statements to explore whether gender differences exist and consider the dual roles of specialty-specific values and gender norms in shaping gender differences in applicant language use.

## Methods

We performed retrospective content analysis of residency personal statements submitted during two academic years, 2014–2015 and 2015–2016, for applicants who intended to enter residency during the following academic year. This study was performed at the University of Wisconsin-Madison, USA, within the Pediatrics Department, during the study period of November 2017 through June 2018.

As part of the application to US medical residencies, graduating medical students submit biographical information, medical school and standardized testing transcripts, a Dean’s Letter known as the Medical School Performance Evaluation (MSPE) detailing the student’s medical school performance and extracurricular involvement, letters of recommendation, a personal statement, and an applicant photograph via the Electronic Residency Application System (ERAS). Each residency program then uses its own program-determined criteria to select which applicants to invite for interview. After interviews are conducted, residency programs rank all interviewees using program-specific scoring systems, based on the different elements of the residency application and the interview, and applicants rank the residency programs at which they interviewed. Finally, the National Residency Matching Program (NRMP), utilizes a mathematical algorithm based on applicant and residency program rankings to “match” applicants into residency positions [36].

While other residency programs may use the personal statement in determining which applicants to invite for interview, our program and many others utilize only the most objective measures of residency qualification and clinical skill from the application (medical school transcripts and standardized testing scores) to determine interviewee selection. Our program then reviews the subjective portions (personal statements, MSPE, and letters of recommendation) after the interview to help determine final applicant rank.

All applications from medical students who interviewed with the pediatrics residency program in the two application cycles were eligible for inclusion, except for current residents whose personal statements were excluded. This population was purposefully chosen for this initial study examining linguistic characteristics of pediatric personal statements to help understand gender differences in the writing of the candidates most likely to be interviewed by pediatric residency programs. Current residents were excluded from this study. Exclusion of current residents was based on collaborative decision-making between the study team, pediatrics residency leadership, and the IRB in effort to reduce the risk of identification of any personal statement author by study team members who work with the residents based on written content of their personal statement, even in the absence of unique identifiers. Additionally, the only demographic information provided to the study team was applicant self-reported gender. Options for applicant gender in ERAS included, “Male,” “Female,” “Decline to answer,” and “No answer.” No applicants in the study pool selected, “Decline to answer” or “No answer” for gender, so only male and female comparisons were performed.

We used the text analysis software, Linguistic Inquiry and Word Count (LIWC), previously used in numerous studies evaluating gender biases in medicine and academia [22, 24, 37, 38]. LIWC is a linguistic analysis tool that aids in the study of cognitive, emotional, and structural aspects of written and verbal speech [39, 40]. The LIWC program searches bodies of text for words and word parts that match its internal dictionary of 6400 words and word stems. Users may also manually add words to the LIWC program dictionary.

LIWC primarily reports data as percentage of word occurrence in text. For example, when examining references to “reward” in text, LIWC output is the percentage of “reward” words (including, “fulfill,” “promote”, and “benefit”) used within the text. For a subset of variables, output is reported instead as a LIWC-validated scaled score, based on standardized scores derived from large comparison samples of linguistic characteristics found in speech and writing across a variety of settings in the general US population [39, 41–44].

### Measures

#### Linguistic dimensions, writing tone

Linguistic dimensions included word count, average words per sentence, and complex word count (defined by words longer than 6 letters). Overall writing tone of personal statements was determined using four variables based on population samples of text across a variety of settings [39, 41–44]. Writing tone variables included: [1] analytic tone (tendency to use formal or informal word choices) [2]; emotional tone (tendency to use of positive or negative emotion words) [3]; clout (tendency to use language expressing expertise or tentativeness); and [4] authenticity (tendency to use language expressing vulnerability or guardedness), as scored by the LIWC program.

#### Agentic & communal language

Given previous findings of gender differences in agentic and communal language for males and females [7, 9, 34, 35], agentic and communal word use were our primary variables of interest. To evaluate agentic and communal language, we selected LIWC dictionary categories used in prior studies evaluating gender differences in residency applications and other social and professional contexts [9, 10, 22, 24, 37, 38]. We also manually added dictionaries for agentic and communal language, described previously in content analysis by Madera, et al. and utilized by Li, et al. in evaluation of letters of evaluation for emergency medicine [24, 37]. Agentic words included “fulfill,” “benefit,” “success,” “achieve,” “think,” “know,” and “confident.” Communal words included “family,” “friend,” “perhaps,” “maybe,” “kind,” and “helpful.” Table 1 includes the list of example dictionary words for each category.

**Table 1:** Dependent Variables Examined Using Linguistic Inquiry Word Count (LIWC) [39]

Category	Example LIWC dictionary words
Agentic Language	
*LIWC dictionary*	
Reward	Fulfill, benefit, opportunity
Risk	Danger, doubt
Power	Superior, bully
Achievement	Success, better
Insight	Think, know
Certainty	Always, never
*Madera* et al *dictionary (manually added)*	Assertive, confident, ambitious, aggressive, dominant, forceful, independent, daring, outspoken, intellectual, earn, gain, do
Communal language	
*LIWC dictionary*	
Social affiliation	Family, friend, children
Family	Daughter, father, brother
Friends	Friend, neighbor
Male references	Boy, his, dad
Female references	Girl, her, mom
Tentativeness	Perhaps, maybe
*Madera* et al. *dictionary (manually added)*	Affectionate, nurturing, helpful, kind, sympathetic, sensitive, agreeable, tactful, interpersonal, warm, caring, tactful, husband, wife, babies, kids, colleagues, they, him, her

### Procedure

All 85 male-authored personal statements that met eligibility criteria for our study were included. In order to avoid over-sampling females (which represented over 75% of the interviewee pool), female-authored personal statements were randomly selected using random number generator to obtain an equal number of personal statements, resulting in 170 personal statements total (85 from males, 85 from females) that were then provided to the study team for analysis in de-identified form.

In the first phase of content analysis, we assessed proper LIWC word categorization for pediatric residency personal statements. Using a sample of 10 personal statements, two authors (JB, AG) independently evaluated LIWC output variables to find words that might commonly occur in personal statements but be categorized inappropriately by LIWC. For instance, “Children’s” (e.g. “Children’s Hospital”) was frequently used as a proper noun and if not excluded in this context, would have been counted inappropriately as a word of social affiliation. Similarly, the words “practice,” “admitted,” and “down” (as in Down Syndrome) have different meaning in medicine as compared to their categorization in LIWC. An investigator (AG) reviewed use of these words in all 170 personal statements in order to ensure appropriate word categorization based on context.

### Analysis

Next, we entered all personal statement texts into the LIWC program and analyzed them using the LIWC dictionary variables for linguistic dimensions; writing tone; agentic language; and communal language. We then analyzed the personal statements using the manually entered dictionaries based on the work of Madera, et al. for agentic and communal language [37]. For each LIWC output variable, two-tailed t-tests (*p* < 0.05) were performed using STATA (STATA Software 15.0) [45] to compare mean percentage of word use and mean LIWC-validated scaled score for male- and female-authored personal statements.

### IRB

The Education and Social/Behavioral Science Institutional Review Board at the University of Wisconsin-Madison deemed this study non-human subjects research (2017–1422) and therefore exempt from its review, given that the personal statements for this study were provided to the study team for analysis without identifiers, and the identities of the writers could not be ascertained.

## Results

A total of 423 applicants interviewed with our pediatrics residency program in 2015 and 2016, of whom 96 (22.7%) identified as male and 327 (77.3%) identified as female. After exclusion of current residents (11 males, 19 females), 393 personal statements were eligible for inclusion and 170 personal statements were ultimately included (43.3% of eligible personal statements).

### Linguistic dimensions, writing tone

Average total word count was 676.4 words (SD = 113.31) with an average of 22.5 words per sentence (SD = 3.83). Complex language accounted for 27.82% of all analyzed personal statement text (SD = 3.68). There were no gender differences in word count (male authors 674.5, SD = 110.51, female authors 678.4, SD = 116.67, *p* = 0.83); words per sentence (male authors 22.25%, SD = 3.72, female authors 22.80%, SD = 3.93, *p* = 0.35); or complex word count (male authors 27.99%, SD = 3.59, female authors 27.64%, SD = 3.79, *p* = 0.54) for males compared to females.

Personal statements scored as highly analytic (LIWC scaled score 81.41, SD = 9.84, range 0–100, higher scores indicating predominantly formal writing) [39] and as demonstrating positive emotional tone (LIWC scaled score 86.44, SD = 15.56, range 0–100, higher scores indicating more frequent use of positive over negative emotion words) [39]. Scores were mid-range in measures of authenticity (LIWC scaled score 58.77 SD = 18.66, range 0–100, higher scores indicating more expressive writing) [39]. Scores were mid-range for clout, suggesting equal balance in using authoritative and tentative language (LIWC scaled score of 51.11 SD = 13.59, range 0–100, higher scores indicating more frequent use of language expressing expertise) [39]. There were no significant differences in LIWC scaled scores for males and females for any of the writing tone variables, *p* > 0.05. See Appendix A for detailed results.

### Agentic language

Males used agentic language expressing the psychological drive of reward more frequently than females, accounting for 1.80% (SD = 0.73) of all words written by males and 1.55% (SD = 0.67) of words written by females, (*p* = 0.02). The range of reward word frequency used by applicants was consistent with the percentage of reward words typically used by the general population in natural speech (1.73%) and expressive writing (1.56%) [39]. Commonly identified words of reward in this study included, “fulfillment,” “rewarding,” and “opportunity.” Table 2 demonstrates excerpts of reward language.

**Table 2:** Example Excerpts of Reward Language in Pediatric Residency Personal Statements

“Through this conversation, I further realized the **rewarding opportunity** presented to pediatricians- using their skills to serve ill children and their families with professionalism, excellence, and compassion.”	
“While **opportunities** to learn, serve, and teach exist in every medical specialty, the ability to do so is particularly salient in pediatrics.”	
“I’m drawn to pediatrics because it incorporates medicine, public health, and social work together in order to provide children with the **best** chance at living a healthy, **successful** life.”	
“There is an immense potential in children [such] that I experience **great fulfillment** in trying to help them **achieve**.”	

Legend: Bolded words indicate reward language.

There were no differences in other language of agency between males and females, including words for risk (male authors 0.45%, SD = 0.82, female authors 0.44%, SD = 0.31, *p* = 0.91), power (male authors 3.37%, SD = 0.95, female authors 3.45%, SD = 0.95 *p* = 0.58), risk (*p* = 0.82), achievement (male authors 3.27%, SD = 1.01, female authors 3.13%, SD = 1.07 *p* = 0.38), insight (male authors 3.19%, SD = 1.01, female authors 3.26%, SD = 1.04, *p* = 0.66), and certainty (male authors 1.24%, SD = 0.59, female authors 1.16%, SD = 0.51, *p* = 0.34). Evaluation of agentic language using the manually entered dictionary for agentic words likewise showed no gender differences in the use of agentic adjectives (assertive, confident, ambitious), (male authors 0.05%, SD = 0.10, female authors 0.03%, SD = 0.07, *p* = 0.30) or orientation (earn, gain, do), (male authors 0.28%, SD = 0.29, female authors 0.23%, SD = 0.23, *p* = 0.21). See Appendix B for detailed results.

### Communal language

There was no statistical difference between male and female authors in the use of any variables measuring communal language. Language of social affiliation accounted for 2.79% words written by males (SD = 1.07), and 2.95% of words written by females (SD = 1.02), (*p* = 0.31), slightly higher than that which is typically found in natural speech (2.06%), expressive writing (2.45%), and blogs (2.20%) for the general population [39]. References to family accounted for 0.99% of text written by males (SD = 0.65) and 1.14% written by females (SD = 0.79), (*p* = 0.20). References to friends accounted for < 0.5% words written by both males and females, (male authors 0.14%, SD = 0.17; female authors 0.11, SD = 0.14; *p* = 0.17). There were no differences in male references (male authors 0.98%, SD = 1.08; female authors 0.98%, SD = 0.99; *p* = 0.99) and female references (male authors 0.81%, SD = 0.91; female authors 0.88%, SD = 0.54; *p* = 0.59).

Tentative language was used with similar occurrence by males and females, accounting for 1.48%, of words written by males (SD = 0.68) and 1.31% of words written by females (SD = 0.57), (*p* = 0.09). Evaluation of communal language using the manually entered dictionary for communal words likewise showed no statistical gender differences in the use of communal adjectives (affectionate, nurturing, helpful, kind), (male authors 0.11%, SD = 0.16; female authors 0.12%, SD = 0.13, *p* = 0.49) or orientation (husband, wife, baby, colleague), (male authors 1.54%, SD = 0.75; female authors 1.62%, SD = 0.75, *p* = 0.48). Table 3 demonstrates excerpts of communal language. See Appendix B for detailed results.

**Table 3:** Example Excerpts of Communal Language in Pediatric Residency Personal Statements

“Returning after a year of working with **children** and **parents** in the **community** emphasized my desire to work as both a pediatrician and a **member** of the **public** health **community**.”	
“I want to be an advocate for my patients, **provide** education and **social** resources, and to be valuable **member** of the medical **team**.”	
“These **visits** reinforce my passion to **help families** in **their** most vulnerable times. Putting **children** and **families** in the best position to thrive requires awareness and **listening**.”	
“Most importantly, by **engaging** my young patients, I can teach **them** that the doctor-patient **relationship** is a true **partnership**.”	

Legend: Bolded words indicate communal language.

## Discussion

Our study found that applicants to pediatrics residency wrote their personal statements with a highly analytical and moderately authentic style with positive emotional tone. Both males and females wrote in a non-assertive way, striking a tone that evenly balanced expertise and tentativeness.

The most notable finding in our study was that males applying to pediatrics used communal language on par with their female counterparts. This finding deviates from typical gender norm expectations, which would predict that males use social language less than females [7–12] and suggests that personal statement language may be partially dictated by the applicant’s perception of specialty-specific values, regardless of gender norms for language use.

Students rotating through pediatrics, for example, are taught that family-centeredness is the gold standard in providing pediatric care [46–48]. Students receive feedback on their family-centered rounding skills and may even be required to attend workshops on optimizing family-centered care [46, 47]. This emphasis and evaluation of the pediatric trainee’s skills in relationship-centeredness demonstrates to the student applicant that communal traits are not only expected but highly valued by faculty in pediatrics. Additionally, mentor physicians helping to review and edit personal statements may encourage this career assimilation as they assist applicants in revising their personal statements.

Moreover, when males represent the minority in a workplace (36.6% male workforce in pediatrics in the US), social and professional pressure may exist that encourages communal behavior and speech (traits more often associated with females in prior studies) [7–11]. Studies by Cejka and Eagly have shown that “to the extent that occupations were female dominated, feminine personality or physical attributes were thought more essential for success; to the extent that occupations were male dominated, masculine personality or physical attributes were thought more essential” [49]. Communal attributes may be highly valued by the male applicants who choose to pursue a career in pediatrics; however males entering pediatrics might also be subject to subtle social and professional pressure to include anecdotes of communal relationships and utilize communal language more frequently than typical gender norms would predict.

The finding that applicants veer from typical linguistic gender norms as a means of describing alignment with specialty-specific values is consistent with the findings of Osman et al. and Ostapenko, et al. which previously examined personal statements for internal medicine and general surgery residencies [34, 35]. While the primary aims of these studies were not to examine the influence of specialty-specific values on personal statement writing, the studies did find that the primary themes described by both male and female applicants were themes of agency, which gender norms would not typically predict for females. Unlike pediatrics, internal medicine and general surgery are male-dominated fields (62.1 and 79.4% male workforces in the US, respectively) [50]. As such, the cultural values of these specialties likely favors an image of agency overall (i.e. academic rigor, technical expertise) [7], implicitly encouraging applicants, regardless of gender, to conform to these expectations.

In our study, we also found that applicants did not completely deviate from gender norms for language use. Males in our study used agentic language of reward more than females, consistent with gender expectations. This finding is also consistent with findings from internal medicine and surgery personal statements in which gender differences in minor themes were found (females described the importance of teamwork and communication, males utilized self-promoting anecdotes and descriptions of technical expertise) [34, 35]. These findings suggest that despite the tendency to appeal to specialty-specific values for the majority of personal statement content, the use of gender normative language (agency for males, communality for females) may persist in subtle ways, demonstrating the potential pervasiveness of gender expectations on the applicant.

There are a few limitations to this study. The first is that although the LIWC program has been validated in social psychology literature and shown to have good reliability in using “marker words” in place of content coding [10], we could have missed words or ideas that would have been otherwise identified if context was considered. Additionally, our study population was drawn from a single US medical school and from only those who interviewed with our program, rather than all applicants. Although our program’s approach to interviewee selection, which utilizes only the most objective parts of the application (scores, transcripts), significantly reduces the risk that our sample selected for a population that demonstrated program-preferred values (eg. communality), this remains possible. Additionally, by focusing on this population, we were able to describe the linguistic characteristics of applicants who would most likely be interviewed at a pediatric US residency program based on objective measures of achievement in US and international medical schools (scores, transcripts). However, we were unable to determine whether certain linguistic characteristics in personal statements are preferred and selected for by reviewers in the interview selection process, which may vary internationally and across cultures. Finally, our study cannot be generalized to other fields of medicine; however, as there remains a current gap in the literature focusing on understanding how language patterns vary by specialty, generalizations across specialties should not be made until additional studies are undertaken specifically examining each specialty.

This is the first study to our knowledge that has characterized the linguistic qualities and gender differences of pediatric residency personal statements and considered ways in which professional specialty choice and gender expectations might dually shape the way an applicant writes. It is important for residency training programs to understand the potential implications of these subtle language findings in personal statement writing for two reasons. First, understanding the social pressures that may influence an applicant’s writing may help training programs determine how and whether to utilize the personal statement in their applicant ranking process, knowing that in addition to its known limitations as a tool to predict future resident success, this subjective element of the application may result in unintended bias in the evaluation of the applicant’s “fit” for the program [28–31]. If residency programs are not aware of the potential for bias in evaluating the personal statement, they may inadvertently exclude highly qualified applicants based on subtle qualities of applicants’ writing that do not reflect their skills as physicians. Additionally, understanding the role of societal gender norms and expectations for communal language use by females and agentic language use by males and how those expectations may shift within a medical specialty will allow educators to consider ways in which these expectations enter the application process, particularly when reviewers are not attuned to their own expectations and bias, as well as other educational settings.

The findings of this study also has potential practical implications for residency programs. Residency programs that utilize the personal statement in their selection process might consider methods of limiting gender bias in evaluation of the personal statement by blinding reviewers to applicant biographical information and photograph or creating other standardized measures of personal statement evaluation and scoring [31]. Additionally, this study sheds light on the potential consideration of whether linguistic analysis could be utilized in the evaluation of personal statements to help programs identify candidates that possess positive interpersonal and professional traits that align with program values. This approach could represent a standardized approach in evaluating personal statements that when applied to all personal statements, indiscriminate of applicant gender, could select for the more intangible applicant interpersonal characteristics that programs may seek (eg. communality in pediatrics). However, programs would have to carefully consider the ways in which utilizing this method could also unintentionally perpetuate systematic or cultural bias, and future studies would be required to investigate validity of this approach in screening applicants.

In order to further understand the way specialty-specific expectations and gender norms enter the residency application process across medical fields and applicant populations, it would be important to perform linguistic analysis of personal statements from a wide array of specialties, including male-dominated fields (orthopedics, urology) and female-dominated fields (obstetrics), as well as differences in highly ranked applicants as compared to lower or unranked applicants to determine if certain linguistic characteristics correlate with applicant ranking. Additionally, future studies could focus on applicants who identify as gender non-conforming (nonbinary, transgender identity), in order to understand the true breadth of gender differences in the application process. Finally, other applicant characteristics, such as ethnicity and country of origin could be included in future analyses. Doing so could provide residency programs with more complete understanding of how gender biases operate across cultures and empower residency programs to optimize diversity, inclusion, and equity in the application process.

## Conclusions

In writing a personal statement for application to pediatric residency programs, applicants may be subject to dual pressures of upholding gender norms while also demonstrating shared professional values with the members of their anticipated profession. While applicants may utilize language that deviates from gender expectations for communality and agency in order to convey their “fit” in their anticipated career, gender normative language likely persists in personal statements in subtle ways. Evaluation of linguistic gender differences in highly ranked and low-ranking applicants, across medical specialties, and applicants with different cultural backgrounds would help expand understanding of how specialty-specific values and gender expectations dually influence the residency selection process and allow for possible gender bias that may persist throughout the physician’s career. With improved understanding of these subtle social processes, we will be better able to provide education about implicit bias and consider ways to reduce bias in the residency selection process, with the ultimate goal of cultivating medical training systems that reduce the negative impact of these biases.

## Data Availability

The datasets generated and/or analysed during the current study are not publicly available due to them containing information that could compromise privacy protections, and restrictions apply to the availability of the data, but data may be available from the corresponding author on reasonable request and if additional permissions are granted.

## Appendix A

**Table 4:** LIWC Scaled Scores for Pediatric Personal Statement Writing Tone Variables

Word category	*All authors* LIWC Score^a^	*Male authors* LIWC Score^a^	*Female authors* LIWC Score^a^	*p* value
*Tone*				
Analytic	81.41 ± 9.84	81.14 ± 9.99	81.66 ± 9.75	*p* = 0.73
Emotional	86.44 ± 15.56	87.10 ± 15.11	85.79 ± 16.07	*p* = 0.58
Clout	51.11 ± 13.59	50.36 ± 13.76	51.86 ± 13.44	*p* = 0.47
Authenticity	58.77 ± 18.66	56.78 ± 19.15	60.77 ± 18.06	*p* = 0.16

^a^LIWC scores range 0–100. Higher scores for analytic thinking, clout, and authenticity indicate higher degrees of use of words demonstrating analytic thinking, clout, and authenticity. Scores > 50 for emotional tone indicate positive emotional tone; scores < 50 indicate negative emotional tone

## Appendix B

**Table 5:** Percentage of Pediatric Personal Statement Text Containing Agentic and Communal Language

Word category	*All authors* Percentage of text (%)	*Male authors* Percentage of text (%)	*Female authors* Percentage of text (%)	*p* value
*Agentic Language,*				
*LIWC dictionary*				
Reward	1.67 ± 0.71	1.80 ± 0.73	1.55 ± 0.67	*p* = 0.02
Risk	0.45 ± 0.30	0.45 ± 0.82	0.44 ± 0.31	*p* = 0.91
Power	3.42 ± 0.95	3.37 ± 0.95	3.45 ± 0.95	*p* = 0.58
Achievement	3.20 ± 1.04	3.27 ± 1.01	3.13 ± 1.07	*p* = 0.38
Insight	3.23 ± 1.02	3.19 ± 1.01	3.26 ± 1.04	*p* = 0.66
Certainty	1.20 ± 0.63	1.24 ± 0.59	1.16 ± 0.51	*p* = 0.34
*Agentic Language,*				
*Madera,* et al. *dictionary*				
Adjectives	0.04 ± 0.09	0.05 ± 0.10	0.03 ± 0.07	*p* = 0.30
Orientation	0.26 ± 0.27	0.28 ± 0.29	0.23 ± 0.23	*p* = 0.21
*Communal Language,*				
*LIWC dictionary*				
Social affiliation	2.87 ± 1.05	2.79 ± 1.07	2.95 ± 1.02	*p* = 0.31
Family	1.06 ± 0.72	0.99 ± 0.65	1.14 ± 0.79	*p* = 0.20
Friends	0.13 ± 0.16	0.14 ± 0.17	0.11 ± 0.14	*p* = 0.17
Male references	0.98 ± 1.03	0.98 ± 1.08	0.98 ± 0.99	*p* = 0.99
Female references	0.84 ± 0.91	0.81 ± 0.91	0.88 ± 0.91	*p* = 0.59
Tentativeness	1.40 ± 0.63	1.48 ± 0.68	1.31 ± 0.57	*p* = 0.09
*Communal Language,*				
*Madera,* et al. *dictionary*				
Adjectives	0.12 ± 0.15	0.11 ± 0.16	0.12 ± 0.13	*p* = 0.49
Orientation	1.59 ± 0.75	1.54 ± 0.75	1.62 ± 0.75	*p* = 0.48
